# Clinical Value of Presepsin in Comparison to hsCRP as a Monitoring and Early Prognostic Marker for Sepsis in Critically Ill Patients

**DOI:** 10.3390/medicina55020036

**Published:** 2019-02-02

**Authors:** Elham A. Hassan, Abeer S. Abdel Rehim, Asmaa O. Ahmed, Hanan Abdullahtif, Alaa Attia

**Affiliations:** 1 Department of Tropical Medicine and Gastroenterology, Faculty of Medicine, Assiut University, Assiut 71111, Egypt; sharafabeer@yahoo.com; 2 Department of Clinical Pathology, Faculty of Medicine, Assiut University, Assiut 71111, Egypt; asomar_12@yahoo.com (A.O.A.); Abdlatif@aun.edu.eg (H.A.); 3 Department of Anesthesia and Intensive Care, Faculty of Medicine, Assiut University, Assiut 71111, Egypt; alaa.atya@med.au.edu.eg

**Keywords:** sepsis, presepsin, hsCRP, in-hospital mortality

## Abstract

*Background and objectives:* Sepsis carries a poor prognosis for critically ill patients, even withintensive management. We aimed to determined early predictors of sepsis-related in-hospital mortality and to monitor levels of presepsin and high sensitivity C reactive protein (hsCRP) during admission relative to the applied treatment and the development of complications. *Materials and Methods:* An observational study was conducted on 68 intensive care unit (ICU) patients with sepsis. Blood samples from each patient were collected at admission (day 0) for measuring presepsin, hsCRP, biochemical examination, complete blood picture and microbiological culture and at the third day (day 3) for measuring presepsin and hsCRP. Predictors of sepsis-related in-hospital mortality were assessed using regression analysis. Predictive abilities of presepsin and hsCRP were compared using the area under a receiver operating characteristic curve. The Kaplan–Meier method was used to estimate the overall survival rate. *Results:* Results showed that the sepsis-related in-hospital mortality was 64.6%. The day 0 presepsin and SOFA scores were associated with this mortality. Presepsin levels were significantly higher at days 0 and 3 in non-survivors vs. survivors (*p* = 0.03 and *p* < 0.001 respectively) and it decreased over the three days in survivors. Presepsin had a higher prognostic accuracy than hsCRP at all the evaluated times. *Conclusions:* Overall, in comparison with hsCRP, presepsin was an early predictor of sepsis-related in-hospital mortality in ICU patients. Changes in presepsin concentrations over time may be useful for sepsis monitoring, which in turn could be useful for stratifying high-risk patients on ICU admission that benefit from intensive treatment.

## 1. Introduction

Sepsis is the life-threatening failure of organs caused by a dysregulated host response toinfection that carries a significant morbidity and mortality [1,2]. It develops in 12.4–27.3% of intensive care unit (ICU) patients [3,4]. Even though there have been new advances in critical care management such as the improvements of diagnostic criteria, the higher index of infection suspicion and improvedantibiotic therapy, the incidence of sepsis is steadily increasing among hospitalized patients where the fatality rates unacceptably remains as high as 30–60%, while the mortality rate of severe sepsis accounts for 30–50% of hospital deaths [5,6,7,8,9].

In this viewpoint, early identification and monitoring of those patients is important to alleviate the risk of multi-organ failure and to decrease mortality. The available scoring systems that could predict hospital outcomes for critically ill patients carry some shortages e.g., Acute Physiology and Chronic Health Evaluation II (APACHEII) and Sequential Organ Failure Assessment (SOFA) scores [10,11]. One of the shortages of the APACHEII scoring system is its complexity, while SOFA is not well known outside the critical care community. A recent quick SOFA scoring system has been proposedin patients with suspected infection, however its assessment strategy has a low sensitivity in ICU patients [12]. Other organ failure scoring systems exist, including systems built from statistical models, but none are in common use [1].

Although, laboratory indexes such as high sensitivity C reactive protein (hsCRP) and procalcitonin are highly specific for sepsis, their levels can be increased in settings other than bacterial infection resulting in false-positive results e.g., autoimmune diseases, tumors, tissue necrosis after ischemic heart attack, severe trauma, invasive surgical procedure and critical burn injuries [13,14,15].

Presepsin (sCD14-ST) is the soluble N-terminal region of the membrane marker/receptor protein CD14, which is released after the host cell activation that follows the recognition of bacterial lipopolysaccharide or other surface bacterial ligands including gram-positive peptidoglycans [16]. Several studies documented the role of presepsin in the diagnosis and prognosis of sepsis where presepsin levels were significantly higher in septic than in non-septic patients or in those with systemic inflammatory response syndrome (SIRS) [6,17]. Moreover, these higher levels were reported in the early stages of sepsis that were correlated with severity [9].

The aim of this study was to identify the early predictors of sepsis-related in-hospital mortality in critically-ill patients during ICU admission, and to assess the clinical value of presepsin and hsCRP monitoring during admission in relation to the applied treatment and to the development of complications.

## 2. Materials and Methods

### 2.1. Study Design

This was an observational cohort study carried out at Assiut University Hospital (AUH), a tertiary-care hospital in Egypt, from June 2017 to March 2018. The study was approved by the Local Ethics Committee of AUH, Egypt, and was conducted in accordance with the previsions of the Declaration of Helsinki. Patients (or relatives of comatose patients) provided informed consent for study participation. The ethical approval code is 17100657 (on date: 12/8/2014).

### 2.2. Study Population

Patients were admitted to ICU of Emergency Department, Assiut University Hospital, Assiut, Egypt. They were diagnosed as having SIRS and consecutively recruited from June 2017 to March 2018.

The diagnosis of SIRS was defined as two or more of the following conditions: (i) temperature >38 °C or <36 °C, (ii) heart rate >90 beats/minute, (iii) respiratory rate >20 breaths/minute or PaCO2 <32 mmHg when on mechanical ventilation, (iv) white blood cell count >12,000/μL or <4000/μL, or an increase in the number of immature band forms (>10%), according to criteria of the ACCP/SCCM Consensus Conference Committee as reported by Bone et al. [18].

Exclusion criteria were age ≤18 years, pregnant women, cancer diseases and patients with immunosuppressive drug treatment, patients with acquired immunodeficiency syndrome, end-stage liver and renal disease.

At the time of ICU admission (Day 0), patients underwent a thorough medical history and physical examination and were observed untildischarge from ICU or death. Sepsis-related in-hospital mortality was assessed where patients were stratified as survivors ornon-survivors.

The SOFA score was assessed at the time of ICU admission (Day 0) to evaluate the severity of critical patients and to evaluate sepsis-related organ dysfunction.

### 2.3. Specimen Collection and Processing

For each patient, 15–20 mL of blood was collected aseptically within the first hours after admission (Day 0) and at the third day (Day 3) of enrolment. Two milliliters of the blood sample were used for plasma separation by centrifugation at 3000 *g* for 10 min and stored at −20 °C (for measurements of presepsin). The remaining sample was used for biochemical examination, complete blood picture, and microbiological culture.

### 2.4. Specimen Examination

Liver function, kidney function, blood picture (total and differential white blood cell count), serum glucose, prothrombin time (PT) and activated partial thromboplastin time (aPTT) were measured.

Estimation of the plasma level of presepsin was done by the chemiluminescent enzyme immunoassay PATHFAST Presepsin (Mitsubishi Chemical Medience Corporation, Japan) using Magtration Technology as per the manufacturer’s instructions. A value >200 pg/mL was considered positive as indicated by the manufacturers.

High sensitivity C-reactive protein (hsCRP) was measured by nephelometry (BN ProSpec^®^ System, Siemens, Munich, Germany).

For blood cultures, each blood sample was incubated and monitored for seven days by BacT/Alert 3D system (bioMérieux, Marcy l’Etoile, France). Blood culture samples with positive signals were processed. Subcultures were prepared on blood agar, chocolate agar and MacConkey agar, at 37 °C and incubated for 24–48 h, and a gram stain film was takenfrom the growth to identify the type of organism (gram-positive or negative, cocci or bacilli).

The identification and antibiograms of growing bacteria were determined with the VITEK 2 Compact 15 (BioMerieux, Marcy l’Etoile, France), a fully automated system for microbial identification and antimicrobial susceptibility testing.

Patients were stratified into two groups: non-bacteremic SIRS patients with persistently negative blood cultures which were excluded from this study and bacteremic patients with positive blood cultures.

### 2.5. Statistical Analysis

All statistical analyses were conducted using Statistical Package for the Social Sciences for Windows version 16 (SPSS Inc., Chicago, IL, USA) and the MedCalc program. Quantitative data are expressed as mean ± standard deviation or median and the interquartile range and were compared using a Student’s *t* test or a Mann–Whitney U-test for normally or abnormally distributed data, respectively. Categorical variables were expressed as a percentage and compared using a chi-squared or Fisher’s exact probability test. Spearman’s rank correlation coefficient (r) was used to find correlations. Significant factors on univariate analysis were considered for inclusion in multiple regression analyses to predict the sepsis-related in-hospital mortality. The receiver operating characteristic curves (ROC) were plotted to measure and compare the performance of presepsin and hsCRP to determine the death risk and to select the best cut-off point at which sensitivity, specificity, positive (PPV) and negative (NPV) predictive values, positive and negative likelihood ratio (+LR, −LR) could be calculated. The Kaplan–Meier method was used to estimate the overall survival rates. All tests were two-tailed and the statistical significance was assessed at <0.05.

## 3. Results

### 3.1. Characteristics of the Studied Patients

During the study period, 90 critically ill patients who met one or more of the SIRS criteria according to American College of Chest Physicians (ACCP)/Society of Critical Care Medicine (SCCM) criteria were admitted to ICU. Twenty-two patients were non-bacteremic SIRS patients with persistently negative blood cultures, and the remaining 68 were bacteremic patients with positive blood cultures.

Those 68 critically ill ICU patients with proven sepsis were included in the study. Their mean age was 35.7 ± 15.1 years and the male sex was predominant (70.6%). The etiology of ICU admission was post traumatic complications. Most of the patients had a gram-negative bacterial infection (67.6%). Sepsis-related organ dysfunctions were evaluated using a SOFA score at admission (7.6 ± 3.1; 3.1–11.8). Of 68 patients, 44 (64.7%) died during hospitalization due to a sepsis-related complication: Intractable multiple organ failure (*n* = 34), coagulation disorders (*n* = 9) and intestinal ischemia (*n* = 1).

The clinical and laboratory data of the studied patients and their subgroups (survivors and non-survivors) at and during admission (Day 3) is summarized below in Table 1.

### 3.2. Assessment of Presepsin and hsCRP in Critically Ill Septic Patients

It was found that the presepsin levels on days 0 and 3 of admission were significantly higher in deceased patients than survivors (*p* = 0.03 and <0.001 respectively, Table 1).

**Figure 1:**
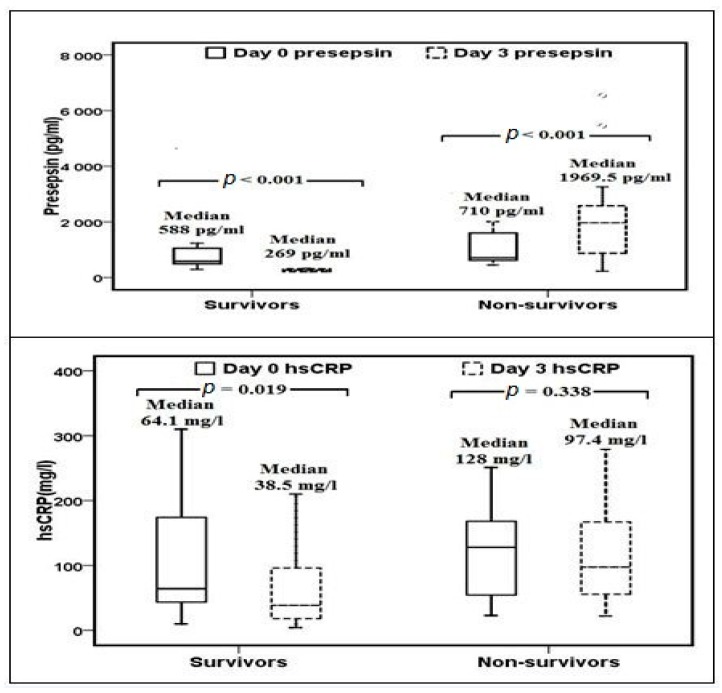
Changes of presepsin and hsCRP levels at days 0 and 3 of admission among survivor and non-survivor septic patients; hsCRP: high sensitivity C-reactive protein.

In addition, Figure 1 showed that in a cohort of survivors at day 3 of admission, the median presepsin values significantly reduced from 588 pg/mL to 269 pg/mL (*p* < 0.001). In contrast, in deceased patients, the median presepsin values significantly increased from 710 pg/mL to 1969.5 pg/mL (*p* < 0.001).

On the other hand, the median hsCRP levels significantly decreased in the survived patients on day 3 (64.1 mg/L to 38.5 mg/L, *p* = 0.019, Figure 1) when compared with deceased patients on day 3 (38.5 mg/L vs. 97.4 mg/L, *p* = 0.015, Table 1). There were no significant differences, however, in hsCRP levels among survivors and non-survivor septic patients on admission (64.1 mg/L vs. 128 mg/L, *p* = 0.488, Table 1) and its levels were not significantly changed from day 0 to day 3 follow-up among deceased patients (128 mg/L vs. 97.4 mg/L, *p* = 0.338) (Figure 1).

Levels of presepsin at admission (day 0) significantly correlated with SOFA (r = 0.745, *p* < 0.001) and with its components; PaO2/FiO2 (r = −0.390, *p* = 0.04), bilirubin (r = 0.601, *p* = 0.001), creatinine (r = 0.551, *p* = 0.001), platelets (r = −0.447, *p* = 0.008) and aPTT (r = 0.508, *p* = 0.002). No significant correlations, however, were found between day 0 hsCRP and these parameters.

Plasma presepsin levels (days 0 and 3) among non-survivors infected with gram-negative bacteria were higher than those infected with gram-positive bacteria, but this was not statistically significant difference. Furthermore, hsCRP levels (days 0 and 3) were not significantly associated with the type of bacteria among deceased patients.

### 3.3. Determination of Risk Factors for Sepsis-Relatedin-Hospital Mortality

Univariate analysis showed that serum bilirubin, creatinine, WBC, presepsin and SOFA on admission (day 0) and hsCRP and presepsin on the third day of admission (day 3) were significantly associated with sepsis-related in-hospital mortality (Table 1).

On multivariate analysis, only SOFA (*p* = 0.03) and presepsin (*p* = 0.04) were found to be independent predictors of sepsis-related in-hospital mortality at admission (Table 2).

### 3.4. Comparison of Predictive Accuracy and Determination of the Best Cut-Off Value of Presepsin and hsCRP for Risk of Sepsis-Related in-Hospital Mortality

**Figure 2:**
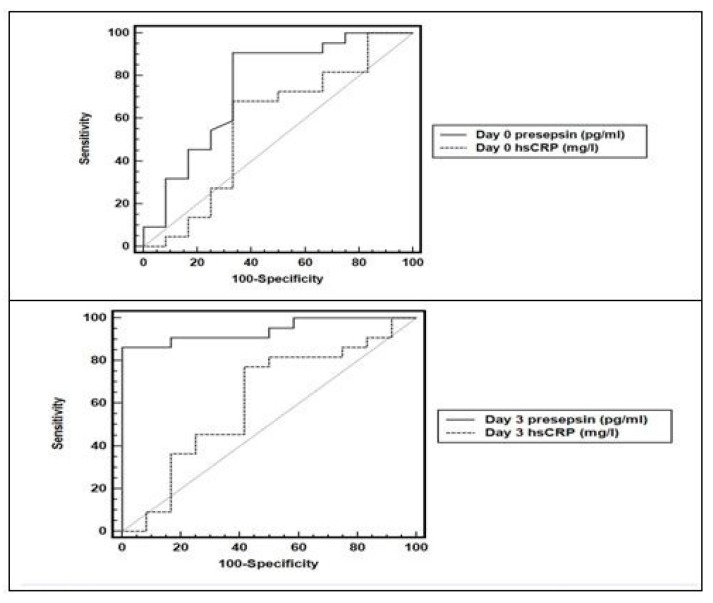
Area under the receiver operating characteristic curve (AUC) of presepsin and hsCRP (on days 0 and 3) where presepsin has higher AUCs in predicting sepsis-related in-hospital mortality in critically ill patients. hsCRP: high sensitivity C-reactive protein.

Based on the ROC curves, presepsin at admission (day 0) had better prognostic accuracy than hsCRP (day 0) for the prediction of sepsis-related in-hospital mortality (Figure 2). Day 0 presepsin yielded higher AUC (0.824) and 95% confidence interval (CI) (0.646–0.955), *p* = 0.03, with 86.4% sensitivity, 89.6% specificity, 93.8% PPV, 78.2% NPV, and 8.3 +LR at cut-off of >607 pg/mL (Table 3).

Furthermore, by using the ROC curve, the ability of the presepsin values to predict sepsis-related in-hospital mortality at follow up (on day 3) revealed higher AUC (0.943) and 95% CI (0.806–0.992) with 90.1% sensitivity, 100% specificity, 100% PPV and 89.8% NPV with a cut-off of >1323 pg/mL (*p* < 0.001) (Figure 2, Table 3).

### 3.5. Determination of the Survival Analysis

**Figure 3:**
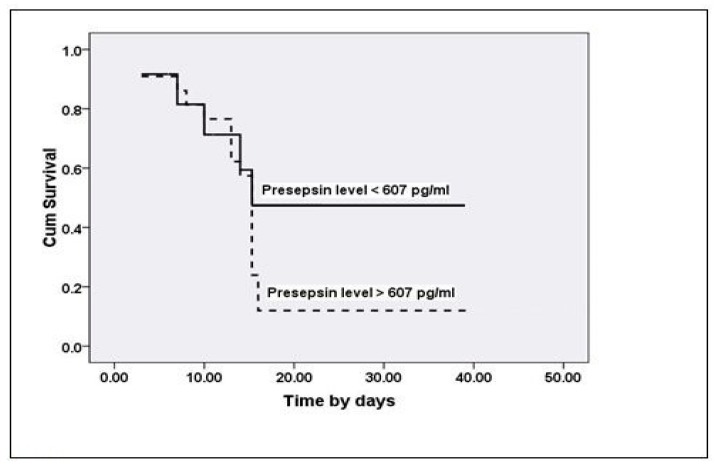
Kaplan–Meier survival curves for day 0 presepsin predicting increased sepsis-related in-hospital mortality with higher values (*p* < 0.05).

Kaplan–Meier-estimated survival curves were generated for patients who fell above and below the cut-off values identified by means of the ROC curves for day 0 presepsin to predict sepsis-related in-hospital mortality (Figure 3). These cut-offs clearly differentiated between patients with different survival times; patients who had higher levels than the cut-off value (>607 pg/mL) had a shortened survival period compared to patients who had lower levels (long rank, *p* = 0.360; Breslow, *p* = 0.576 and Tarone-Ware, *p* = 0.047). On the other hand, no significant changes were found in survival period with changes in levels of day 0 hsCRP.

## 4. Discussion

The life-threatening condition of sepsis is a great challenge amongst the critical care population. In this study, sepsis-related in-hospital mortality among critically ill patients was 64.6%, which was higher than the 18.4% to 60% reported in previous studies [5,6], and lower than that reported in India (85%) [19]. These variations of sepsis-related mortality are likely dependent on multiple factors involving variations in the definition of sepsis. We intended in this study to evaluate the prognostic value of presepsin in critically ill patients and its impact on sepsis-related in-hospital mortality in comparison to hsCRP.

In this study, presepsin levels were associated with sepsis-related in-hospital mortality within the first three days of ICU admission and it was found that their levels were significantly higher in non-survivor septic patients than survivors, which was in agreement with earlier studies [20,21]. Masson et al. [22] reported that increasing presepsin levels within the first week of hospitalization predicted ICU and 90-day mortality.

In the present study, it was demonstrated that presepsin levels within the first week had better prognostic accuracy than hsCRP in the prediction of sepsis-related in-hospital mortality. They had the highest AUC value (0.943) on day 3 rather than on day 0, whichmay anticipate poor response to treatment with exaggerated inflammatory response. This was in accordance with Liu et al. [20] who showed that presepsin had a better prognostic capacity for predicting short and long-term mortality in septic patients than interleukin 6, CRP and procalcitonin. Masson and colleagues found that AUCs for presepsin on days 1, 2, and 7 for predicting in-hospital mortality were 0.96, 0.70, and 0.74 respectively [22].

Identifying cut-off values of presepsin at admission (607 pg/mL) and on the third day (1323 pg/mL) which had high sensitivity (86.4 and 90.1%), specificity (89.6 and 100%) and PPV (93.8 and 100%) in the current results indicated that presepsin may be a good predictor for mortality that necessitates the construction of more intensive measures to reduce the high mortality rates. Moreover, patients with higher values than the day 0 presepsin cut-off value had a short survival rate. Klouche et al. [23] who showed that presepsin was a predictor of ICU mortality in septic patients at a cut-off value of 1925 pg/mL.

In this study, there was an increase in presepsin levels in deceased patients infected with gram-negative bacteria compared tothose infected with gram-positive bacteria, but with no statistical significance. This finding was compatible with Romualdo and colleagues who reported that gram-negative were associated with the highest levels of presepsin without achieving any statistical significance, indicating that presepsin levels were not dependent on the type of bacterial organism [24].

We found that presepsin levels in survivors declined significantly on day 3 compared to its admission levels and compared to non-survivors. These findings were consistent with previous studies, indicating the possibility of using the presepsin levels to monitor the efficacy of antimicrobial therapy [22,25].

It has been demonstrated that presepsin is generated as the body response to bacterial infection and its production is induced by phagocytosis of bacteria [26]. It has inhibitory activity on both innate immune cells (macrophages) and on T and B cells. So, its production is restricted to infection rather than the degree of inflammation [16], unlike CRP whichincreases in cases of SIRS even without bacterial infection [20]. Furthermore, elevated presepsin levels in deceased patients may be partially attributed to sepsis-related complications, e.g., acute kidney injury [27].

Also, day0 presepsin levels were significantly correlated with the SOFA score and aPTT and inversely correlated with the platelets count suggesting the role of presepsin as a predictor of ongoing organ dysfunction. These findings were in agreement with Behnes et al. [28] except that there was no correlation with the platelets count. Masson et al. [22] revealed that higher presepsin on day 1 was closely associated with a higher incidence of subsequent new organ failures. Moreover, Ishikura et al. [29] reported that presepsin levels were higher in patients with lower platelet counts, reflecting a possible relationship between presepsin and coagulation disorders. Masson et al. [22] revealed that “presepsin appears to be a good marker of the host response, and its higher levels, independently of the type of infection per se, may indicate a loss of infection compartmentalization, or a state of immunoparalysis, leading to a spreading of the related inflammatory reaction, and of the innate immune host response, which may ultimately lead to multiple organ failure and death”.

C-reactive protein is widely used in the critical care setting, and its value as a prognostic marker is proven in many diseases including sepsis [30,31]. Similar to Endo et al. [32] we demonstrated that survivors had hsCRP levels that decreased significantly over time.

In our study, the measurement of hsCRP at admission was not associated with mortality in septic patients, however its measurement after 72 h was significantly higher in non-survivors but with a low predictive capability (AUC = 0.737). In addition, at a cut-off value >67 mg/L, hsCRP had 54.6% sensitivity, 75% specificity, 80% PPV and 47.4% NPV. These findings agreed with Thiem et al. [31] who reported that the initial CRP level did not predict mortality in hospitalized patients with community-acquired pneumonia. Silvestre et al. [33] concluded that the initial CRP was not an adequate marker for the prognosis of sepsis patients. In contrast, Hogarth et al. [34] reported that non-survivors had a significantly higher median CRP concentration on admission than survivors. Ho et al. [30] showed that a high CRP level was an independent risk factor of mortality. El-Shafiea et al. [35] reported that CRP levels did not show any significant difference between survivors and non-survivors on days 0, 2, or 4.

C-reactive protein is an acute phase reactant, so its persistence may represent an emerging subclinical nosocomial infection or unresolved inflammation in critically ill patients [30]. In addition, its concentration correlated with organ dysfunction in those patients [36,37]. Lobo et al. [36] reported that in a heterogeneous group of critically ill patients, the concentrations of CRP fall as organ dysfunction resolves in survivors, but remains elevated in non-survivors. On the other hand, its elevation may be related to non-infectious conditions e.g., severe trauma as was the case in our patients, resulting in false-positive results [13,14,15].

There were some limitations of this study. It was a single-center small sample sized study. It was limited to study bacteremia patients with positive cultures, however nearly two thirds of those patients with sepsis never had positive blood cultures. In addition, it was focused on hospital deaths, however many sepsis deaths may have occurred after hospital discharge. So, large multicenter cohort studies will be emphasized to confirm these findings and to identify post-discharge prognosis.

## 5. Conclusions

In comparison with hsCRP, presepsin could be an early predictor for sepsis-related in-hospital mortality in critically ill patients. Moreover, changes in presepsin concentrations over time may be useful for the monitoring of sepsis, which may aid in further improvement of the quality of care in sepsis patients and in the further reduction of their short-term mortality. Overall, these findings may offer a useful strategy to stratify high-risk patients on ICU admission who would benefit with intensive treatment.

## Figures and Tables

**Table 1:** Demographic and clinical characteristics of the studied critically ill patients with sepsis (survivors and non-survivors).

	Total (*n* = 68)	Survivors (*n* = 24)	Non-Survivor (*n* = 44)	*p*
Age (years; Mean ± SD)	35.7 ± 15.1	34.7 ± 15.3	36.3 ± 15.2	0.771
Sex (M/F)	48/20 (70.6/29.4%)	20/4 (83.3/16.7%)	28/16 (63.6/36.4%)	0.228
SOFA score	7.6 ± 3.1	6.3 ± 3.2	8.9 ± 2.9	0.015
Type of organisms (Gram+/−ve)	22/46 (32.4/67.6%)	0/24 (0/100%)	22/22 (50/50%)	0.05
Glucose (mmol/L; mean ± SD)	6.9 ± 2	7.2 ± 2.2	6.8 ± 1.9	0.581
Urea (mmol/L; median, range)	6.1 (1.3–30.4)	8.2 (4.2–16.1)	5.8 (1.3–30.4)	0.118
Creatinine (µmol/L; median, range)	84.5 (35.9–663.8)	103.5 (35.9–296)	378.8 (37–663.8)	0.03
Albumin (g/dL; mean ± SD)	2.6 ± 0.7	2.7 ± 0.7	2.6 ± 0.7	0.556
Total bilirubin (µmol/L; median, range)	10.7 (3–85)	9.4 (3–33.5)	21.4 (8.5–85)	0.04
AST (U/L; median, range)	102 (19–989)	122.5 (31–597)	84.5 (19–989)	0.261
ALT (U/L; median, range)	58.5 (14–298)	111.5 (16–298)	49.5 (14–194)	0.094
INR	1.6 ± 0.2	1.2 ± 0.2	1.8 ± 0.2	0.400
Hemoglobin (g/dL; mean ± SD)	10 ± 2.3	10.2 ± 1.6	9.9 ± 2.5	0.736
Platelets (×10^9^/L; median, range)	193 (20–625)	196.5 (53–625)	187 (20–275)	0.548
WBC (×10^9^/L; mean ± SD)	12.9 ± 5.6	10.8 ± 4.7	15 ± 6.4	0.03
WBC at follow-up (×10^9^/L; mean ± SD)	11 ± 4.9	10.2 ± 3.9	11.5 ± 5.4	0.462
hsCRP (mg/L; median, range)	101.5 (9.8–384)	64.1 (9.8–384)	128 (22.8–380)	0.488
hsCRP at follow-up (mg/L; median, range)	66 (4–279)	38.5 (4–210)	97.4 (22–279)	0.015
Presepsin (pg/mL; median, range)	690 (294–4965)	588 (294–4965)	810.5 (453–4879)	0.03
Presepsin at follow-up (pg/mL; median, range)	721 (210–6540)	269 (210–323)	1969.5 (232–6540)	<0.001

*p* < 0.05 = significant; ALT: alanine aminotransferase; AST: aspartate aminotransferase; hsCRP: high sensitivity C-reactive protein; INR: international normalized ratio; SOFA: The Sequential Organ Failure Assessment; WBC: white blood cell

**Table 2:** Multiple regression analysis of risk factors predicting sepsis-related in-hospital mortality in the study critically ill patients at admission (day 0).

	Odds Ratio (95% CI)	*p*
Type of organisms (Gram+/−ve)	0.9 (0.8–1.03)	0.342
SOFA score	1.3 (0.8–2)	0.03
Serum creatinine	1 (0.8–1.4)	0.499
Serum total bilirubin	1.1 (0.9–1.9)	0.549
WBCs	0.7 (0.4–1.1)	0.683
hsCRP	1.03 (1–1.1)	0.092
Presepsin	1 (0.9–1.02)	0.04

*p* < 0.05 = significant; hsCRP: high sensitivity C-reactive protein; SOFA: The Sequential Organ Failure Assessment; WBCs: white blood cells.

**Table 3:** Diagnostic accuracy of prognostic parameters to predict sepsis-related in-hospital mortality with the best predictive cut-offs.

	AUC 95% CI	SEN (%)	SPE (%)	PPV (%)	NPV (%)	+LR	−LR	Accuracy (%)
Day 0 Presepsin (>607 pg/mL)	0.824 (0.775–0.955)	86.4	89.6	93.8	78.2	8.3	0.2	87.5
Day 3 Presepsin (>1323 pg/mL)	0.943 (0.806–0.992)	90.1	100	100	89.8		0.1	93.6
Day 0 hsCRP (>58 mg/L)	0.576 (0.395–0.743)	72.7	50	72.7	50	1.5	0.5	64.7
Day 3 hsCRP (>67 mg/L)	0.737 (0.558–0.872)	54.6	75	80	47.4	2.2	0.6	61.8

AUC: area under the curve; hsCRP: high sensitivity C-reactive protein; SEN: sensitivity; SPE: specificity; PPV: positive predictive value; NPV: negative predictive value; +LR: positive likelihood ratio; −LR: negative likelihood ratio.

## References

[1] Singer M., Deutschman C.S., Seymour C.W., Shankar-Hari M., Annane D., Bauer M., Bellomo R., Bernard G.R., Chiche J., Coopersmith C.M. (2016). The Third International Consensus Definitions for Sepsis and Septic Shock (Sepsis-3). JAMA.

[2] Seymour C.W., Liu V.X., Iwashyna T.J., Brunkhorst F.M., Rea T.D., Scherag A., Rubenfeld G., Kahn J.M., Shankar-Hari M., Singer M. (2016). Assessment of Clinical Criteria for Sepsis: For the Third International Consensus Definitions for Sepsis and Septic Shock (Sepsis-3). JAMA.

[3] Angus D.C., Sirio C.A., Clermont G., Bion J. (1997). International comparisons of critical care outcome and resource consumption. Crit. Care Clin..

[4] Linde-Zwirble W.T., Angus D.C. (2004). Severe sepsis epidemiology: Sampling, selection, and society. Crit. Care.

[5] Kaukonen K.M., Bailey M., Suzuki S., Pilcher D., Bellomo R. (2014). Mortality related to severe sepsis and septic shock among critically ill patients in Australia and New Zealand, 2000–2012. JAMA.

[6] Dombrovskiy V.Y., Martin A.A., Sunderram J., Paz H.L. (2007). Rapid increase in hospitalization and mortality rates for severe sepsis in the United States: A trend analysis from 1993 to 2003. Crit. Care Med..

[7] Kumar G., Kumar N., Taneja A., Kaleekal T., Tarima S., McGinley E., Jimenez E., Mohan A., Khan R.A., Whittle J. (2011). Nationwide trends of severe sepsis in the 21st century (2000–2007). Chest.

[8] Vincent J.L., Rello J., Marshall J., Silva E., Anzueto A., Martin C.D., Moreno R., Lipman J., Gomersall C., Sakr Y. (2009). International study of the prevalence and outcomes of infection in intensive care units. JAMA.

[9] Levy M.M., Dellinger R.P., Townsend S.R., Linde-Zwirble W.T., Marshall J.C., Bion J., Schorr C., Artigas A., Ramsay G., Beale R. (2010). The Surviving Sepsis Campaign: Results of an international guideline-based performance improvement program targeting severe sepsis. Intensive Care Med..

[10] Knaus W.A., Draper E.A., Wagner D.P., Zimmerman J.E. (1985). APACHE II: A severity of disease classification system. Crit. Care Med..

[11] Ferreira F.L., Bota D.P., Bross A., Melot C., Vincent J.L. (2001). Serial evaluation of the SOFA score to predict outcome in critically ill patients. JAMA.

[12] Machado F.R., Assunção M.S., Cavalcanti A.B., Japiassú A.M., Azevedo L.C., Oliveira M.C. (2016). Getting a consensus: Advantages and disadvantages of Sepsis 3 in the context of middle-income settings. Rev. Bras. Ter. Intensiva.

[13] Wacker C., Prkno A., Brunkhorst F.M., Shankar-Hari M., Annane D., Bauer M., Bellomo R., Bernard G.R., Chiche J.D., Coopersmith C.M. (2013). Procalcitonin as a diagnostic marker for sepsis: A systematic review and meta-analysis. Lancet Infect. Dis..

[14] Kibe S., Adams K., Barlow G. (2011). Diagnostic and prognostic biomarkers of sepsis in critical care. J. Antimicrob. Chemother..

[15] Au-Yong A. (2012). Towards evidence-based emergency medicine: Best BETs from the Manchester Royal Infirmary. BET 2: C-reactive protein in the diagnosis of bacteraemia. Emerg. Med. J..

[16] Shozushima T., Takahashi G., Matsumoto N., Kojika M., Okamura Y., Endo S. (2011). Usefulness of presepsin (sCD14-ST) measurements as a marker for the diagnosis and severity of sepsis that satisfied diagnostic criteria of systemic inflammatory response syndrome. J. Infect. Chemother..

[17] Memar M.Y., Baghi H.B. (2019). Presepsin: A promising biomarker for the detection of bacterial infections. Biomed. Pharmacother..

[18] Bone R., Balk R., Cerra F., Dellinger R.P., Fein A.M., Knaus W.A., Schein R.M., Sibbald W.J. (1992). Definitions for sepsis and organ failure and guidelines for the use of innovative therapies in sepsis. The ACCP/SCCM consensus conference committee. American college of chest physicians/society of critical care medicine. Chest.

[19] Chatterjee S., Bhattacharya M., Todi S.K. (2017). Epidemiology of Adult-population Sepsis in India: A Single Center 5 Year Experience. Indian J. Crit. Care Med..

[20] Liu B., Chen Y.X., Yin Q., Zhao Y.Z., Li C.S. (2013). Diagnostic value and prognostic evaluation of presepsin for sepsis in an emergency department. Crit. Care.

[21] Masson S., Caironi P., Fanizza C., Thomae R., Bernasconi R., Noto A., Oggioni R., Pasetti G.S., Romero M., Tognoni G. (2015). Circulating presepsin (soluble CD14 subtype) as a marker of host response in patients with severe sepsis or septic shock: Data from the multicenter, randomized ALBIOS trial. Intensive Care Med..

[22] Masson S., Caironi P., Spanuth E., Panigada M., Sangiorgi G., Fumagalli R., Mauri T., Isgrò S., Fanizza C. (2014). Presepsin (soluble CD14 subtype) and procalcitonin levels for mortality prediction in sepsis: Data from the albumin Italian outcome sepsis trial. Crit. Care.

[23] Klouche K., Cristol J., Devin J., Gilles V., Kuster N., Larcher R., Amigues L., Corne P., Jonquet O., Dupuy A.M. (2016). Diagnostic and prognostic value of soluble CD14 subtype (Presepsin) for sepsis and community acquired pneumonia in ICU patients. Ann. Intensive Care.

[24] Romualdo L.G., Torrella P.E., González M.V., Sánchez R.J., Holgado A.H., Freire A.O., Acebes S.R., Otón M.D. (2014). Diagnostic accuracy of presepsin (soluble CD14 subtype) for prediction of bacteremia in patients with systemic inflammatory response syndrome in the Emergency Department. Clin. Biochem..

[25] Spanuth E., Ebelt H., Ivandic B.T., Werdan K., Renz H., Tauber R. (2012). Diagnostic and prognostic value of presepsin (soluble CD14 subtype) in emergency patients with early sepsis using the new assay PATHFAST Presepsin. Advances in Clinical Chemistry and Laboratory Medicine.

[26] Limongi D., D’Agostini C., Ciotti M. (2016). New sepsis biomarkers. Asian Pac. J. Trop. Biomed..

[27] Nakamura Y., Hoshino K., Kiyomi F., Kawano Y., Mizunuma M., Tanaka J., Nishida T., Ishikura H. (2018). Comparison of accuracy of presepsin and procalcitonin concentrations in diagnosing sepsis in patients with and without acute kidney injury. Clin. Chim. Acta.

[28] Behnes M., Bertsch T., Lepiorz D., Trinkmann F., Brueckmann M., Borggrefe M., Hoffmann U. (2014). Diagnostic and prognostic utility of soluble CD14 subtype (presepsin) for severe sepsis and septic shock during the first week of intensive care treatment. Crit. Care.

[29] Ishikura H., Nishida T., Murai A., Nakamura Y., Irie Y., Tanaka J., Umemura T. (2014). New diagnostic strategy for sepsis-induced disseminated intravascular coagulation: A prospective single-center observational study. Crit. Care.

[30] Ho K.M., Lee K.Y., Dobb G.J., Webb S.A. (2008). C-reactive protein concentration as a predictor of in-hospital mortality after ICU discharge: A prospective cohort study. Intensive Care Med..

[31] Thiem U., Niklaus D., Sehlhoff B., Stückle C., Heppner H.J., Endres H.G., Pientka L. (2009). C-reactive protein, severity of pneumonia and mortality in elderly, hospitalised patients with community-acquired pneumonia. Age Ageing.

[32] Endo S., Suzuki Y., Takahashi G., Shozushima T., Ishikura H., Murai A., Nishida T., Irie Y., Miura M., Iguchi H. (2014). Presepsin as a powerful monitoring tool for the prognosis and treatment of sepsis: A multicenter prospective study. J. Infect. Chemother..

[33] Silvestre J., Povoa P., Coelho L., Almeida E., Moreira P., Fernandes A., Mealha R., Sabino H. (2009). Is C-reactive protein a good prognostic marker in septic patients?. Intensive Care Med..

[34] Hogarth M.B., Gallimore R., Savage P., Palmer A.J., Starr J.M., Bulpitt C.J., Pepys M.B. (1997). Acute phase proteins, C-reactive protein and serum amyloid A protein, as prognostic markers in the elderly inpatient. Age Ageing.

[35] El-Shafiea M.E., Taemaa K.M., El-Hallaga M.M., Kandeel A.M.A. (2017). Role of presepsin compared to C-reactive protein in sepsis diagnosis and prognostication. Egypt. J. Crit. Care Med..

[36] Lobo S.M., Lobo F.R., Bota D.P., Lopes-Ferreira F., Soliman H.M., Mélot C., Vincent J.L. (2003). C-reactive protein levels correlate with mortality and organ failure in critically ill patients. Chest.

[37] Castelli G.P., Pognani C., Meisner M., Stuani A., Bellomi D., Sgarbi L. (2004). Procalcitonin and C-reactive protein during systemic inflammatory response syndrome, sepsis and organ dysfunction. Crit. Care.

